# Injuries related to bicycle accidents: an epidemiological study in The Netherlands

**DOI:** 10.1007/s00068-018-1033-5

**Published:** 2018-10-15

**Authors:** Livia E. V. M. de Guerre, Said Sadiqi, Loek P. H. Leenen, Cumhur F. Oner, Steven M. van Gaalen

**Affiliations:** 1 Department of Surgery, University Medical Center Utrecht, Utrecht, The Netherlands; 2 Department of Orthopaedics, University Medical Center Utrecht, HP G05.228, P.O. Box 85500, 3508 GA Utrecht, The Netherlands; 3 Departement of Orthopaedics, Diakonessenhuis Utrecht/Zeist, Utrecht/Zeist, The Netherlands

**Keywords:** Bicycle, Bike, Traffic accident, Traffic injury, E-bike, Epidemiology

## Abstract

**Background:**

This study aims to analyze the incidence and outcomes of bicycle-related injuries in hospitalized patients in The Netherlands.

**Methods:**

Bicycle accidents resulting in hospitalization in a level-I trauma center in The Netherlands between 2007 and 2017 were retrospectively identified. We subcategorized data of patients involved in a regular bicycle, race bike, off-road bike or e-bike accident. The primary outcomes were mortality rate and incidence of multitrauma. Secondary outcomes were differences between bicycle subcategories. Independent risk factors were identified using multivariable logistic regression. All variables with a *p* value < 0.20 in univariable analysis were entered in multivariable analysis.

**Results:**

We identified 1986 patients. The mortality rate after emergency room admission was 5.7%, and 41.0% were multitraumas. A higher age, multitrauma and cerebral haemorrhages were independent risk factors for in hospital mortality. Independent risk factors found for multitrauma were a higher age, two-sided trauma, e-bike accidents and cerebral haemorrhage.

**Conclusion:**

Bicycle accidents resulting in hospitalization have a high mortality rate. Furthermore, a high incidence of multitrauma, fractures and cerebral haemorrhages were found. Considering the increasing incidence of bicycle accident victims needing hospital admission, new and more efficient prevention strategies are essential.

## Introduction

Cycling is a popular means of transportation and leisure activity with many health and environmental advantages. However, simultaneously with the increasing popularity of cycling, concerns for road safety have grown. A recent Australian study estimated that per 1000 km cycled 0.29 crashes occur [1]. Also, a Dutch study showed that in 2012, 31% of the lethal traffic accidents and 59% of the traffic accident victims treated in the emergency room were cyclists [2]. Parallel to the regular city bicycles, other bicycle types have gained popularity and sales of race bikes, off-road bicycles and e-bikes have grown [3]. Each subtype is known for its specific end users and preferred cycling environment but little is known about the differences in injury risks with specific morbidity and mortality.

Biking is rooted in Dutch culture and every year approximately one million bicycles are sold in The Netherlands [3]. Compared to other European countries, The Netherlands has a higher prevalence of cycling as a mode of transport but also a higher incidence of severe bicycle crash injuries [4]. Yearly costs of bicycle-related accidents in The Netherlands are estimated to be 402 million euros [2].

Several studies have been conducted to assess bicycle-related injuries in the general population; however, studies regarding bicycle-related injuries treated in the emergency room are lacking. A clear understanding of the epidemiology of this group will permit better emergency care and aid to implement effective injury prevention strategies. Therefore, the objective of this study is to gain insight in the epidemiology of bicycle accidents in a level-I trauma center in The Netherlands, as well as the identification of trauma patterns and factors that may predict the outcomes.

## Materials and methods

### Patients

All patients who were admitted to the University Medical Center Utrecht (a large level-I trauma center in The Netherlands) after emergency care following a bicycle accident between 2007 and 2017 were retrospectively identified in a well-established trauma care database.

Data were obtained for patients that were involved in a regular bicycle, race bike, off-road bike or e-bike accident. The data collected included patient characteristics (age, gender), trauma mechanism (vehicle, one-sided or two-sided injury mechanism, helmet protection), injury characteristics [diagnosis, fractures, haematomas, injury severity score (ISS)] and mortality. The injuries were classified as minor or multitrauma according to the ISS score. In line with the available literature, the cut-off point for a multitrauma patient was settled as > 15 for the abbreviated injury severity (AIS)-98 classification system, or > 12 for the AIS-08 system [5].

### Outcomes

The primary outcome measures of the study were mortality between the arrival at the emergency department and hospital discharge, and the occurrence of multitrauma. Secondary outcomes were the differences between regular bicycles, race bikes, off-road bikes and e-bikes.

### Statistical analysis

Statistical analyses were performed using SPSS. Normally distributed and non-normally distributed continuous variables were expressed as mean (± SD) or median (range). Categorical data was presented as total counts and percentages. The incidence of all accidents was calculated for each subgroup. The relation between survival and the baseline variables were evaluated by Chi square test, Fisher exact test or Mann–Whitney *U* test. A multivariate logistic regression analysis was performed to identify independent predictors for survival. All variates with a *p* value < 0.2 in the univariate analysis were included in the multivariate analysis. Associations were considered significant when the *p* value was < 0.05.

## Results

### Patient characteristics

**Fig. 1:**
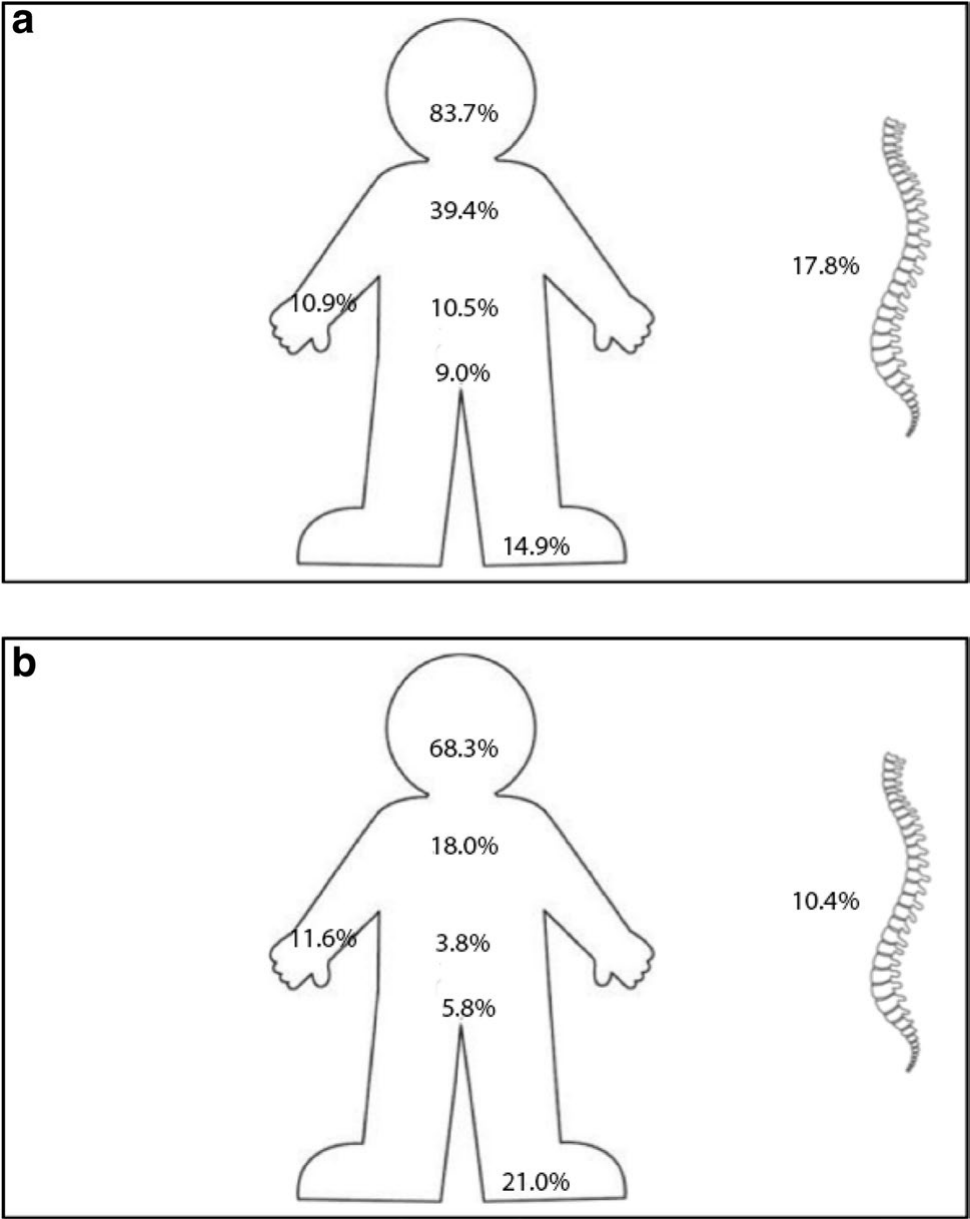
**a** Anatomical distribution of the injuries in multitrauma patients. **b** Anatomical distribution of the injuries in patients with minor trauma. Since multiple patients had more than one body part affected the total is > 100%

A total of 1986 bicycle-related accidents were identified in the database, out of which 1655 concerned regular bicycle accidents (83.3%), 195 race bikes (9.8%), 78 off-road bicycles (3.9%) and 58 e-bikes (2.9%) (Table 1). Of all patients presented in the emergency department, 41.0% were multiply injured. The recorded mortality was 5.7%. The mean age at diagnosis was 45 years, 61.1% of the patients were male and the majority did not wear a helmet (92.5%). The accidents were one-sided in 49.6% of the cases and 73.0% had at least one fracture (Table 2). As shown in Fig. 1, 83.7% of the patients with a multitrauma suffered from a head or neck injury, 39.4% had thoracic trauma, 10.5% abdominal injuries, 9.0% pelvic injuries, 10.9% upper extremities, 14.9% lower extremities and 17.8% spine injuries. In patients with a minor trauma, significantly less patients had a head or neck injury (68.3%), thoracic trauma (18.0%), abdominal injuries (3.8%), pelvic injuries (5.8%) and spinal injuries (10.4%); however, significantly more had a lower extremity injury (21.0%) and a similar percentage had upper extremities injury (11.6%). Table 3 shows a stratification of the sustained fractures, with the most prevalent being facial fractures (28.2%), skull fractures (19.8%) and rib fractures (17.2%). Cerebral haemorrhages were common: 16.6% suffered from a subdural haematoma and 17.0% from a subarachnoid haemorrhage (SAH). Less common were epidural haematomas (5.4%) and intracerebral haemorrhage (5.5%).

**Table 1:** Incidence of bicycle traumas per subgroup

Bicycle subgroup	*N* (%)
Regular bicycle	1655 (83.3)
Race bike	195 (9.8)
Off-road bicycle	78 (3.9)
E-Bike	58 (2.9)
Total	1986

**Table 2:** Baseline characteristics

	*N* (%)
Gender	
Male	1213 (61.1)
Female	767 (38.6)
Missing	6 (0.3)
Age	Mean 45.0 (SD 24.1)
Accident	
One-sided	985 (49.6)
Two-sided	1001 (50.4)
ISS	Mean 13.6 (SD 10.6)
Deceased	
No	1860 (93.6)
Yes	114 (5.7)
Missing	12 (0.6)
Helmet	
No	1838 (92.5)
Yes	148 (7.5)
Fracture	
None	536 (27.0)
At least 1	1450 (73.0)
Cerebral haemorrhages	
Epidural	95 (5.4)
Subdural	295 (16.6)
Subarachnoidal	304 (17.0)
Intracerebral	96 (5.5)
Aftermath	
Home	5 (< 1)
Hospital ward	1080 (54.4)
Transferred out	83 (4.1)
MCU	275 (13.8)
ICU	358 (18)
OR	178 (9.0)
Deceased in the emergency room	3 (2.0)
DOA	3(2.0)
Missing	1 (< 1)

*SD* standard deviation, *DOA* death on arrival, *ICU* Intensive Care Unit, *MCU* Medium Care Unit, *OR* operating room, *Aftermath* admission location after emergency room treatment

**Table 3:** Incidence of fractures

Fracture	*N* (%)
Skull	393 (19.8)
Skull base	87 (4.4)
Facial	561 (28.2)
Spine fractures	252 (12.7)
Humerus	58 (2.9)
Lower arm	73 (3.7)
Scapula	48 (2.4)
Sternal	21 (1.1)
Clavicle	168 (8.5)
Rib	342 (17.2)
Pelvis	127 (6.4)
Femur	140 (7.0)
Lower leg	185 (9.5)

### Risk factors for mortality

Age, gender, multitrauma, non-regular bicycle accidents, one- or two-sided accidents and cerebral haemorrhages were identified as possible risk factors for mortality. These risk factors were included in the multivariate logistic regression model. The analysis identified a higher age, multitrauma and cerebral haemorrhages as independent risk factors for mortality.

### Risk factors for multitrauma

Independent risk factors for multitrauma were higher age, two-sided trauma, bicycle type and cerebral haemorrhage. Univariate analysis for multitrauma accidents identified age, e-bike accidents, one- or two-sided accidents and occurrence of cerebral haemorrhages as possible risk factors.

### Trends

**Fig. 2:**
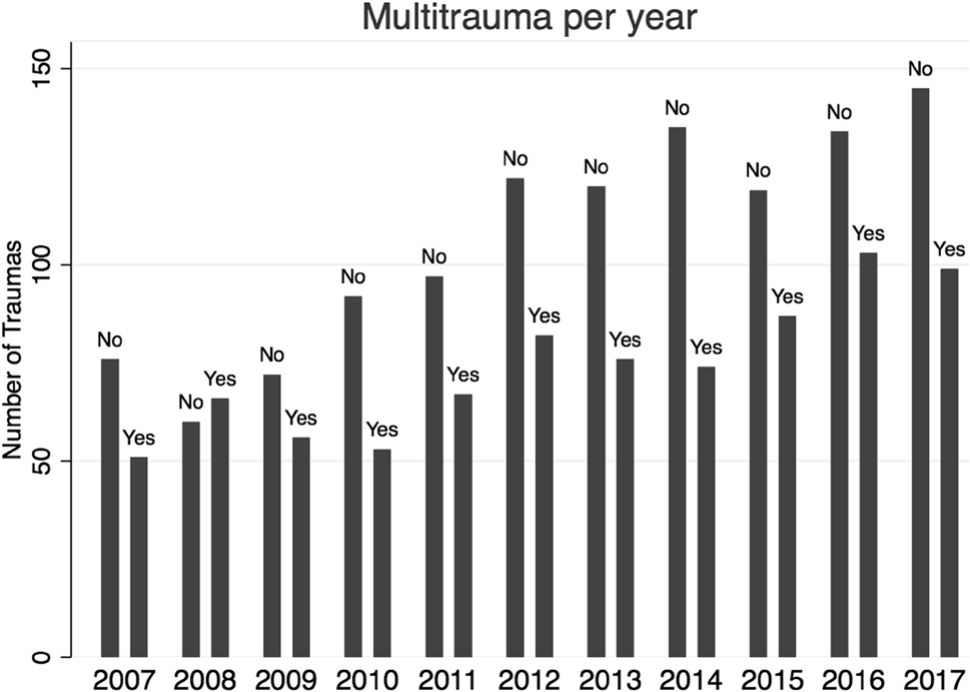
Incidence of minor and multitrauma bicycle traumas per year

An increase in the total number of accidents was seen between 2009 and 2012. From 2012 onwards, the incidence of bicycle traumas has been relatively stable. The incidence of minor traumas increased over the years, whereas the multitrauma incidence remained relatively stable (Fig. 2).

### Bicycle subcategories

Compared to patients sustaining trauma with regular bicycles, race bike trauma patients were older, more often male and the accidents were more often one-sided. Off-road bike accidents have significantly increased in recent years compared to regular bicycles, these were more often one-sided, and patients were more often male and younger. A specific bicycle subgroup concerns e-bikes. Compared to classic bicycles, e-bike trauma patients are older, mortality is more common, more accidents include multitrauma, and the number of accidents has increased significantly in recent years (Table 4).

**Table 4:** Bicycle subgroups

	Regular bicycle	Race bike	Off-road	E-bike
Gender				
Male	944 (57.0)	167 (85.6)	74 (94.9)	28 (48.3)
Female	706 (42.7)	27 (13.9)	4 (5.13)	30 (51.7)
Missing	5 (0.3)	1 (0.5)		
Age	43.9 (SD 25)	50.8 (SD 15.2)	39.6 (SD 18.3)	64.3 (SD 15.8)
Accident				
One-sided	762 (46.0)	114 (58.5)	75 (95.2)	34 (58.6)
Two-sided	893 (54.0)	81 (41.5)	3 (3.8)	24 (41.4)
Helmet				
No	1645 (99.4)	93 (47.7)	43 (55.1)	57 (98.3)
Yes	10 (0.6)	102 (52.3)	35 (44.9)	1 (1.7)
Cer. hemorrhage				
Epidural	83 (5.0)	6 (3.1)	3 (3.9)	3 (5.2)
Subdural	254 (15.4)	17 (8.7)	9 (11.5)	15 (25.9)
SAB	260 (15.7)	17 (8.7)	6 (7.7)	21 (36.2)
Intracerebral	84 (5.1)	3 (1.5)	é (2.6)	7 (12.1)
Multitrauma				
No	997 (63.3)	111 (61.3)	50 (66.7)	27 (58.7)
Yes	579 (36.7)	70 (38.7)	25 (33.3)	19 (41.3)
Deceased				
No	1544 (93.3)	189 (96.9)	77 (98.7)	50 (86.2)
Yes	102 (6.2)	4 (2.1)	1 (1.3)	7 (12.1)
Missing	9 (0.5)	2 (1.0)		1 (1.7)

*SD* standard deviation

### Helmet protection

In the small subgroup of cyclists wearing a helmet (7.5%), 2.0% of the patients died versus 6.0% of the patients who did not wear a helmet. However, this difference was not significant. When wearing a helmet, significantly less patients had head and neck injuries, subdural bleedings, intracerebral bleedings, skull fractures and skull base fractures.

## Discussion

The aim of this study was to gain insight in the incidence and outcomes of bicycle-related injuries in hospitalized patients in The Netherlands.

Bicycle injuries resulting in hospitalization were characterized by a high mortality of 5.7%, and a considerably high multitrauma incidence of 41.0%. In a Dutch study investigating bicycle-related traumatic brain injuries, 4% of the cyclists treated at the emergency department deceased in the hospital due to their multiple injuries [6]. The somewhat higher incidence found in our study could be explained by the more severely injured patient population as we only took into account patients who were admitted to the hospital ward after their treatment at the emergency department. In the present study, 73.0% of the patients presented with at least one fracture, while 16.6% and 17.0% suffered from subdural and subarachnoid haemorrhages, respectively. Injuries to the head and thorax were the most common.

Increased age, multitrauma and cerebral haemorrhages were found to be independent risk factors for mortality. For multitrauma, additionally two-sided trauma and e-bike accidents were found as independent risk factors. Previous studies reported not wearing a helmet, increased age and alcohol consumption as risk factors for bicycle crash mortality [2, 7]. A systematic review showed the association between bicycle helmet use and reduced odds of head injury, serious head injury, facial injury and fatal head injury [8]. Our results indicate that the prevalence of helmet wearing in The Netherlands for cyclists remains low. When wearing a helmet this is associated with less head and neck injuries. Furthermore, cerebral haemorrhages are a risk factor for both multitrauma and mortality. These results support that promoting to wear bicycle helmets is an important safety strategy opportunity for Dutch legislators.

Recent research concerning the increasingly popular e-bikes has raised many concerns [9]. In The Netherlands, 27.6% of the total number of fatal bicycle accidents in 2017 were e-bike accidents [10]. In the present study, we found a high mortality and morbidity rate in an older patient group. Possible contributing factors making e-bikes more prone to severe traffic accidents are the speed difference between cyclists and e-bikers using the same traffic lanes, increased risk taking behaviour and misperception of the e-biker’s approaching speed [11]. In this group, especially the older patients show increased use of anticoagulation drugs which makes (more than in the other bicycle categories) wearing helmets essential in the prevention of specifically head related injuries. A first step to decrease severe e-bike accidents was recently made when a new traffic law in The Netherlands made wearing a helmet compulsory in e-bikes surpassing the speed of 25 km/h (speed pedelecs). This followed EU legislation as defined in the white Paper “Rules and Regulations on electric cycles in the EU” categorizing this subcategory of e-bikes as a similar mode of transportation as a L1e-B moped [12]. For this category, compulsory helmet wear for moped was applied. Unfortunately the white paper stated that none of the EU member states have imposed helmet usage on adult users of conventional bicycles consequently exempting the 25 km/h–250 kW pedelecs from compulsory helmet wear. However, increased road user awareness, increased distinctiveness from other bicycles and compulsory helmet wear for all e-bike categories would be needed to reverse the alarming increase in both morbidity and mortality related to e-bike accidents as found in this study.

Hartog et al. estimated that the beneficial effect of cycling due to increased physical activity results in 9 times more gain in life years than the loss from inhaled air pollution and traffic accidents [13]. However, bicycle crashes are still significant contributor to traffic accident-related mortality and morbidity, while often being considered as preventable. Therefore, stronger injury prevention strategies are needed such as education by promoting safety measures and to increase awareness concerning upcoming new (often faster) bicycle subtypes. Secondly, stricter traffic laws may be needed in The Netherlands to stop the current negative trend of increased accidents.

This study has several limitations. The patient group only represents a percentage of all bicycle-related injuries, as only the patients admitted after emergency room care were included. The minor injuries not requiring hospital admission and on site fatal bicycle accidents are not represented in this study creating somewhat a selection bias. It is also conceivable that the incidence of accidents with race bikes, off-road bicycles and e-bike might be much higher, since not all of these accidents will be registered as such but as regular bicycle accidents instead at the emergency room. Furthermore, this is a regional study and our results might, therefore, not apply to other regions with different bicycle-related infrastructure, traffic laws and cycling popularity. However, since the investigated trauma center is one of the largest in The Netherlands, we expect the results to be representative for other Dutch trauma regions. A previous study showed that bicycle crash prevalence and severity in The Netherlands is among the highest in Europe [4]. Therefore, the results of this study might not be comparable to countries with different cycling cultures. Historically, the Dutch adult cyclists seems to be rather reluctant in voluntary use of helmets on regular bicycles but the data from this study should emphasize that the class L1e-A 25 km/h–250 kW pedelec is not a regular bicycle. Further data collection would be required to better understand influencing factors leading to bicycle accidents such as the crash mechanism, speed, infrastructure, alcohol or smartphone use, and more complete data regarding protective gear and the type of bicycle [7].

In conclusion, this study investigated the epidemiology of bicycle accidents in a large level-I trauma center and found a high mortality rate, many multitrauma cases, a high incidence of fractures and cerebral haemorrhages. Considering the increasing incidence of bicycle crash victims needing hospital admission, prevention strategies such as protective gear, better infrastructure and more strict traffic laws are essential. Furthermore, more extensive national databases should be implemented to enable more specific research and gain new insights. The authors make a strong recommendation for stronger legislation on the use of protective helmets especially when e-bikes are involved in the elderly population.
